# Treatment of stage 3 Coats’ disease by endolaser photocoagulation via a two-port pars plana nonvitrectomy approach

**DOI:** 10.1007/s00417-015-2984-4

**Published:** 2015-03-21

**Authors:** Xuan Cai, Peiquan Zhao, Qi Zhang, Haiying Jin

**Affiliations:** Department of Ophthalmology, Xinhua Hospital affiliated to Shanghai Jiaotong University School of Medicine, No. 1665, Kongjiang Road, Shanghai, China 200092

**Keywords:** Coats’ disease, Endolaser, Anti VEGF, Vitrectomy

## Abstract

**Background:**

To evaluate the effectiveness of endolaser photocoagulation by a two-port pars plana nonvitrectomy approach for treating Coats’ disease with shallow exudative retinal detachment.

**Methods:**

This study included 24 patients (23 boys with an age range of 2–17 years, and one girl, age 6 years) with stage 3 Coats’ disease (25 eyes) from December 2012 and May 2014 at a single center. All of the 25 eyes were complicated with serous or total retinal detachment and received none-vitrectomized endolaser: two (23- or 25-gauge) incisions were routinely made 3 mm posterior to the corneal limbus and a laser was applied directly on the abnormal blood vessels. Additional treatments included subretinal fluid drainage (five eyes), intravitreal triamcinolone injection (seven eyes), and intravitreal anti-vascular endothelial growth factor (VEGF) injection (17 eyes). Best-corrected visual acuity, intraocular pressure, and fundus and abnormal vascular changes were recorded to determine therapeutic effects.

**Results:**

Twenty-four out of the 25 treated eyes (96 %) had retina reattached. The number of treatment sessions differed case by case (1–5 sessions, average 1.96) and the time to full treatment of retinal reattachment was 4 months in average. One patient (4 %) presented with retinal redetachment. Five (20 %) eyes received further laser treatment with indirect ophthalmoscope and four eyes (16 %) presented with total retinal detachment at their first visits received consecutive treatments. At the end of the follow-up period (mean, 10.08 months), telangiectasias of 24 (96 %) eyes were resolved and no severe complications occurred.

**Conclusions:**

Endolaser photocoagulation by a two-port pars plana nonvitrectomy approach is an effective treatment for advanced Coats’ disease with serous retinal detachment. The long-term safety of the approach needs further investigation.

## Introduction

In 1908, George Coats [1] described an ocular entity characterized by unilateral retinal vascular abnormalities and retinal exudation usually in boys. Coats’ disease is associated with excessive production of yellowish intraretinal and subretinal exudates [2] and can cause retinal detachment and severe visual loss [3]. Its common signs are decreased visual acuity, strabismus, and leukocoria [4].

Shields et al. [5] proposed the most recent classification with the following stages: stage 1, telangiectasia only; stage 2, telangiectasia and exudation (2A, extrafoveal exudation; 2B, foveal exudation); stage 3, exudative retinal detachment (3A, subtotal; 3B, total); stage 4, total detachment and secondary glaucoma; and stage 5, advanced end-stage disease.

Multiple modalities have been employed to treat Coats’ disease, including diathermy, laser photocoagulation, cryotherapy, subretinal fluid drainage, scleral buckling surgery, pars plana vitrectomy, and intravitreal anti-vascular endothelial growth factor (VEGF) therapy [6–8]. Treatment is aimed at destroying abnormal vasculature and aneurysmal dilations. Nucc et al. first reported selective photocoagulation and young Coats’ patients responded quickly to laser treatment [9]. In advanced cases with serous retinal detachment, laser photocoagulation may not be able to reach the vessels, leading to persistence of subretinal fluid and necessitating multiple courses of treatment [10]. Eyes with advanced Coats’ disease and total retinal detachment may require pars plana vitrectomy with internal or external drainage of subretinal fluid, laser photocoagulation, and silicone oil tamponade.

The purpose of this retrospective study was to assess the effectiveness of endolaser photocoagulation by a two-port pars plana nonvitrectomy approach for treating Coats’ disease with moderate-to-severe serous retinal detachment.

## Materials and methods

Medical records of 24 patients (25 eyes) with stage 3 Coats’ disease diagnosed from December 2012 to May 2014 at a single center were reviewed. All of them presented with serous or total retinal detachment that could not be reached by regular laser photocoagulation and thus undertook minimally invasive operations of endolaser photocoagulation. Some cases were treated combined with drainage of the subretinal fluid, intravitreal triamcinolone injection or anti-VEGF injection. Patient consent was obtained.

Clinical information, including birth history, age, gender, family history, medical history, and systemic and other ocular anomalies, was assessed. All patients routinely underwent indirect ophthalmoscopy. Wide-angle retina photography (RetCam; Clarity Medical Systems, Pleasanton, CA, USA) was performed in patients younger than 5 years and fundus photography (Optos 200Tx; Optos, Dunfermline, Scotland, UK) was performed in older cooperative patients.

**Fig. 1:**
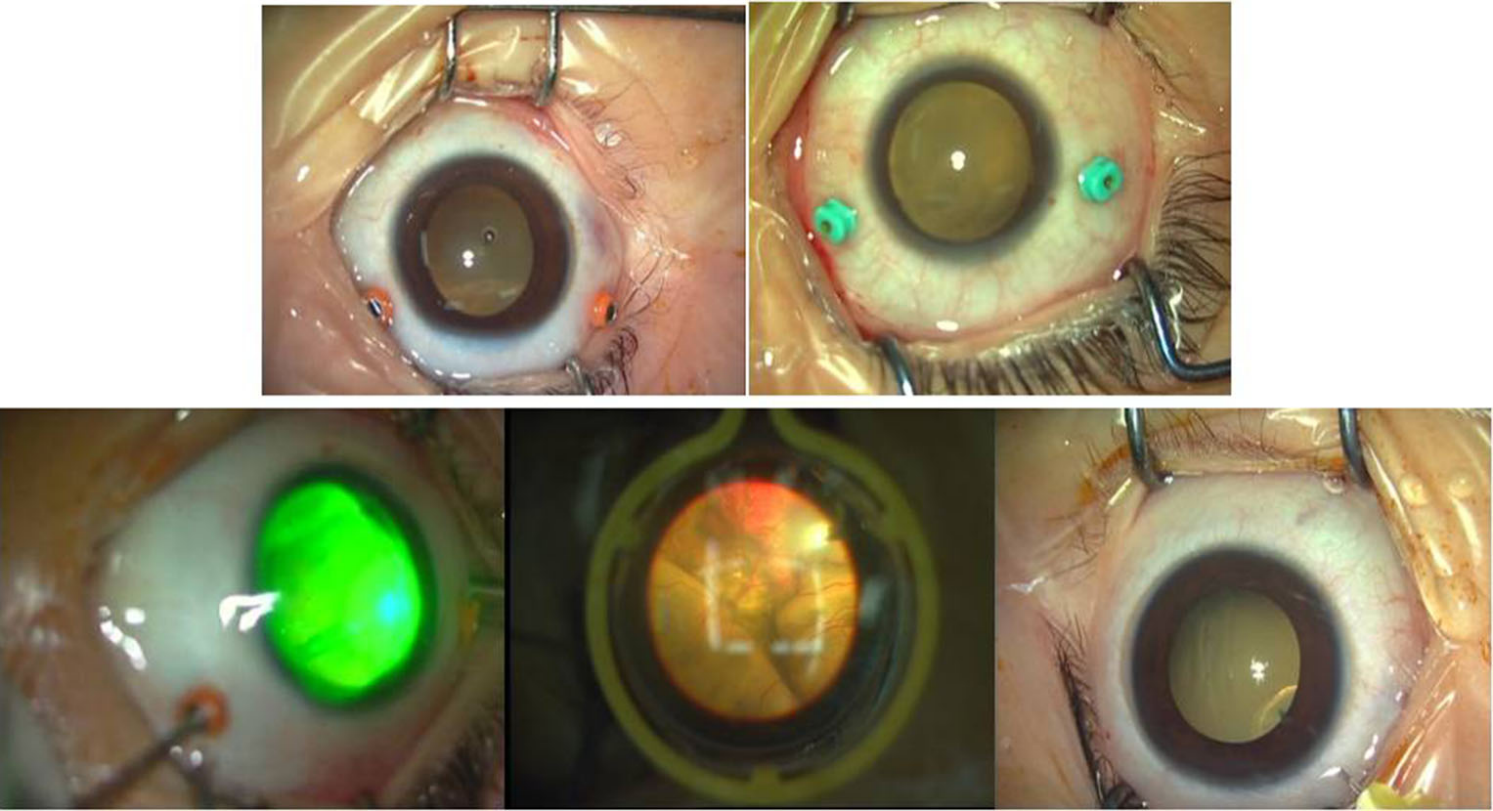
Illustration of the surgical procedure of a 4-year-old boy with stage 3A Coats’ disease (OS). *Top left* Two 23-gauge incisions made 3 mm posterior to the corneal limbus. *Top right* Two 25-gauge incisions made 3 mm posterior to the corneal limbus. *Bottom left* Endolaser ablation of telangiectasias. *Bottom center* Endolaser in the noncontact wide-field viewing system. *Bottom right* Anti-vascular endothelial growth factor (VEGF) injection

**Fig. 2:**
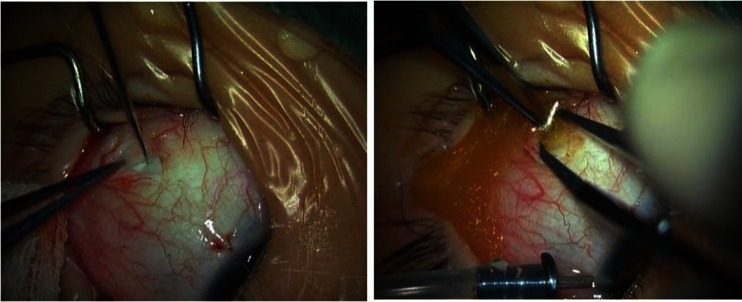
Drainage of subretinal fluid of a 2-year-old boy with stage 3B Coats’ disease (OS)

All surgical procedures were performed under general anesthesia by the same experienced surgeon. Two 23- or 25-gauge incisions were made 3 mm posterior to the corneal limbus and the retina was examined. The surgical procedure commenced with the examine of the retinal vessels, followed by laser directly on the entire network of telangiectatic vessels and the injection of viscoelastics afterwards to minimize vitreous traction at the side ports (Figs. 1 and 2). Telangiectasias were ablated with a 532-nm green endolaser; the power was adjusted according to the retinal reaction to laser spots. A noncontact wide-angle viewing system was used to visualize the fundus. Endolaser therapy was discontinued when whitening of telangiectatic vessels was observed.

Additional treatments such as intravitreal triamcinolone or anti-VEGF injection and further surgery were performed based on findings of fundus fluorescein angiography, conditions during endolaser photocoagulation, and postoperative outcomes. Therapeutic effects were determined according to the patients’ visual acuity, intraocular pressure, fundus and abnormal vascular changes.

All the patients were born at full term or late preterm, with a birth weight above 2,500 g. None had a related family history. The study population comprised 23 boys (95.83 %) and one girl (4.17 %); patient age ranged from 2 to 17 years (mean, 6.21 years). Patients were divided into three groups according to the severity of the disease and previous treatment regimens and the data are presented in Table 1.

**Table 1:** Patient information

Group	Patient	Eye	Gender	Age at presentation (years)	Follow-up duration (months)
A	1	OS	M	3	18
	2	OD	M	2	16
	3	OD	M	8	14
	4	OD	M	17	14
	5	OU	M	3	15
	6	OS	M	11	7
	7	OS	M	5	6
	8	OD	M	5	4
	9	OD	M	3	1
B	1	OS	M	13	18
	2	OS	M	9	17
	3	OD	M	5	16
	4	OS	M	9	16
	5	OD	M	2	14
	6	OS	M	3	12
	7	OD	M	6	4
	8	OS	M	5	4
	9	OD	M	9	3
	10	OD	M	6	3
	11	OD	M	9	2
C	1	OS	M	4	20
	2	OU	M	3	15
	3	OS	M	2	11
	4	OD	M	4	4
	5	OS	F	6	3

Group A (nine eyes) received laser photocoagulation and/or cryopexy before, however, the treatment above could not stop the vascular abnormalities, thus these patients received the endolaser. Group B (11 eyes) was initiated with endolaser treatment regimen due to the presence of serous retinal detachment. Patients in group C (five eyes) were in stage 3B, received external drainage of subretinal fluid plus non-vitrectomized vitrectomy. Additionally, triamcinolone and anti-VEGF were injected according to the condition of abnormal vessels. Visual acuity, intraocular pressure, eye position, slit-lamp microscope, indirect ophthalmoscope, and color fundus imaging were followed up (average, 10.08 months, range, 1–20 months).

## Results

At the end of the follow-up period, 24 (96 %) eyes had the retina reattached, telangiectasias were resolved, and no severe complications occurred. In groups A and B, the exudation involved 3.25 quadrants (range, 2–4). Regular laser therapy under indirect ophthalmoscopy was barely effective on the abnormal vessels with exudative retinal detachment and serous retinal detachment and endolaser could shorten the period of recovery. The average sessions to full treatment of active telangiectasia and retinal reattachment was 4 months (range, 1–10 months); only one (4.17 %) eye progressed to retinal detachment due to lack of follow-up. Each eye received 1–5 sessions of treatments (average, 1.96 times) (Table 2). Increased intraocular pressure in one (4.17 %) eye after surgery was controlled with eye drops.

**Table 2:** Patient treatments

Group	Patient	Range of exudation (quadrant)	Treatments (sessions)
A	1	2	2
	2	4	2
	3	3	2
	4	3	4
	5	4	4
	6	2	1
	7	4	3
	8	2	1
	9	2	3
B	1	4	1
	2	2	1
	3	3	1
	4	4	1
	5	4	1
	6	4	2
	7	4	1
	8	4	1
	9	2	2
	10	4	1
	11	4	1
C	1	4	2
	2	4	5
	3	4	3
	4	4	2
	5	4	2

Seven and 17 eyes required subsequent intravitreal triamcinolone and anti-VEGF injections, respectively. These injections were not associated with endophthalmitis or systemically observed complications. Among the 17 patients who would cooperate with visual examination, five (29.41 %) had improved visual acuity at the end of the treatment session.

**Fig. 3:**
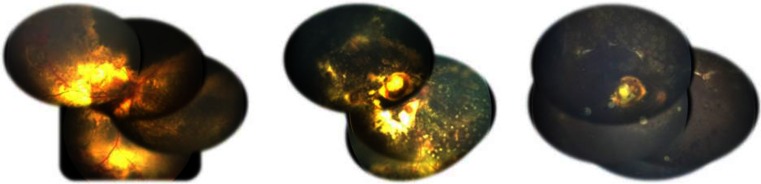
Fundus views of a 2-year-old boy with stage 3A Coats’ disease (OD). *Left* Preoperative view. *Center* Postoperative view during anti-vascular endothelial growth factor (VEGF) injection. *Right* Postoperative view at 13 months

**Fig. 4:**
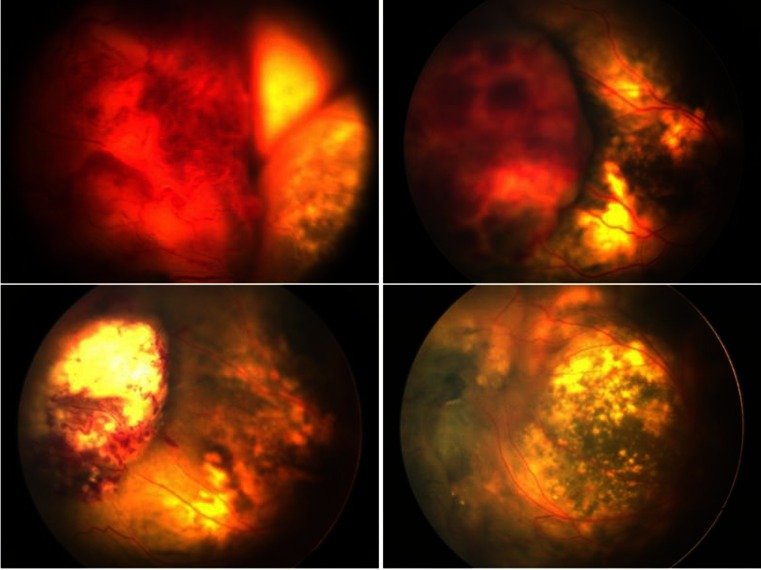
Fundus views of a 3-year-old boy with stage 3B Coats’ disease (OD). *Top left* Preoperative view. *Top right* View at 1 week after SRF drainage. *Bottom left* View at 2 months after endolaser. *Bottom right* View at 2 months after supplementary laser

Because of recurrence of miliary aneurysms, two (22.22 %) eyes in group A and two (18.18 %) eyes in group B had consecutive regular laser ablation at 1 to 6 months (average, 2.25 months) afterward the endolaser treatment. Most exudation was absorbed with a flat retina until the last follow-up (Fig. 3). The five patients in group C who presented with total retinal detachment, undertook drainage of the subretinal fluid combined with anti-VEGF injection, received two-port endolaser ablation and regular laser or other treatments afterwards (Fig. 4). Among them, one eye developed fibrosis in the peripheral retina 4 months after endolaser treatment, so three-port pars plana vitrectomy was performed and the retina finally reattached after three sessions without obvious telangiectasia and the exudation absorption. No enucleation was ultimately necessary.

## Discussion

The treatment of Coats’ disease depends mainly on its severity. The overall aims of treatment in mild disease are ablation of abnormal retinal vasculature, preservation of vision, and prevention of retinal detachment [11]. In advanced cases, the aim is to save the eyeball. In our study, we performed the two-port endolaser with the help with noncontact wide-angle viewing system and fluorescein fundus angiography to guarantee the treatment.

Laser ablation remains the mainstay of treatment. In the present study, vision improved in 29.41 % of the eyes. Schefler et al. [12] showed that 50 % of the patients with Coats’ disease can retain useful vision with aggressive repetitive diode laser therapy even if the disease is advanced at presentation. The difference in outcomes can be explained by the disease severity: the present study included patients with stage 3 Coats’ disease with local or total exudative retinal detachment. According to our experience, this stage is considered less responsive to laser therapy under indirect ophthalmoscopy and the prognosis is worse than those of stages 1 and 2. Further, exudation in the macular area took longer to absorb than peripheral retinal exudation and vision of eyes with macular exudation was not satisfactory in the study.

In this study, we provided a new choice of treating stage 3 Coats’ disease. Yoshizumi et al. [13] and Kranias and Krebs [14] reported successful management of advanced Coats’ disease with pars plana vitrectomy and air–fluid exchange. Susskind et al. used PPV, including a modified technique of exocryotherapy applied after fluid–air exchange to reduce associated side effects and improve the rehabilitation process of advanced Coats’ disease patients [15]. In the present study, stage 3B Coats’ disease was treated by subretinal fluid drainage, endolaser photocoagulation, and anti-VEGF injection. Cryotherapy and silicone oil tamponade was not performed to avoid the damage to the eyeball and the need for secondary surgeries. In two cases, endolaser photocoagulation was performed immediately after subretinal fluid drainage to minimize treatment sessions. The other patients underwent the procedure 1 week after drainage.

To our knowledge, this is the first time the two-port pars plana nonvitrectomy approach for treating Coats’ disease has been reported in the literature. The proposed technique has several advantages. First, it resolves the problem associated with laser ablation in eyes with serous retinal detachment where telangiectasias cannot be reached by regular laser ablation. We applied a laser directly to telangiectasia within the retina without targeting the RPE to create thermal injury. Second, it is non-vitrectomized and is much less invasive than three-port pars plana vitrectomy. Third, when combined with peripheral retinal examination, the procedure could be more thorough and efficient, so the number of treatment sessions could be reduced. To minimize vitreous traction at the side ports, injection of viscoelastics was performed afterwards and turned out to be an effective measure. Shields et al. [5] reported that carefully selected treatment can anatomically stabilize eyes or improve Coats’ disease in 76 % of the cases. The present treatment yielded better results: 96 % of the eyes showed retinal reattachment and no active telangiectasias.

Bevacizumab and ranibizumab should be used cautiously in patients with Coats’ disease to avoid vitreoretinal fibrosis and tractional retinal detachment [16]. A Taiwanese study [17] showed that cryotherapy combined with intravitreal bevacizumab injection in severe cases of exudative retinal detachment carries the risk of vitreoretinal fibrosis and tractional retinal detachment. In the present study, only one eye developed vitreoretinal fibrosis 4 months after anti-VEGF injection, which was in group C and the fibrosis might be connected with the serious disease or the treatments received. Extended serial follow-up is important to evaluate the safety of this treatment and usage of anti-VEGF injection. Supplemental treatment is required for new or recurrent lesions.

## Conclusions

Endolaser photocoagulation by two-port pars plana nonvitrectomy is a safe and effective approach in treating advanced Coats’ disease with serous retinal detachment. Further investigation of this promising technique is warranted.
